# Supplementary material for: Construction of Multiple Guide RNAs in CRISPR/Cas9 Vector Using Stepwise or Simultaneous Golden Gate Cloning: Case Study for Targeting the FAD2 and FATB Multigene in Soybean
Source: Plants (Basel). 2021 Nov 22;10(11):2542. doi: 10.3390/plants10112542 (PMC8622832; doi:10.3390/plants10112542)
Supplement: Supplementary file 1 [file plants-10-02542-s001.zip › Supplementary data_2nd Revision.pptx]

## Slide 1
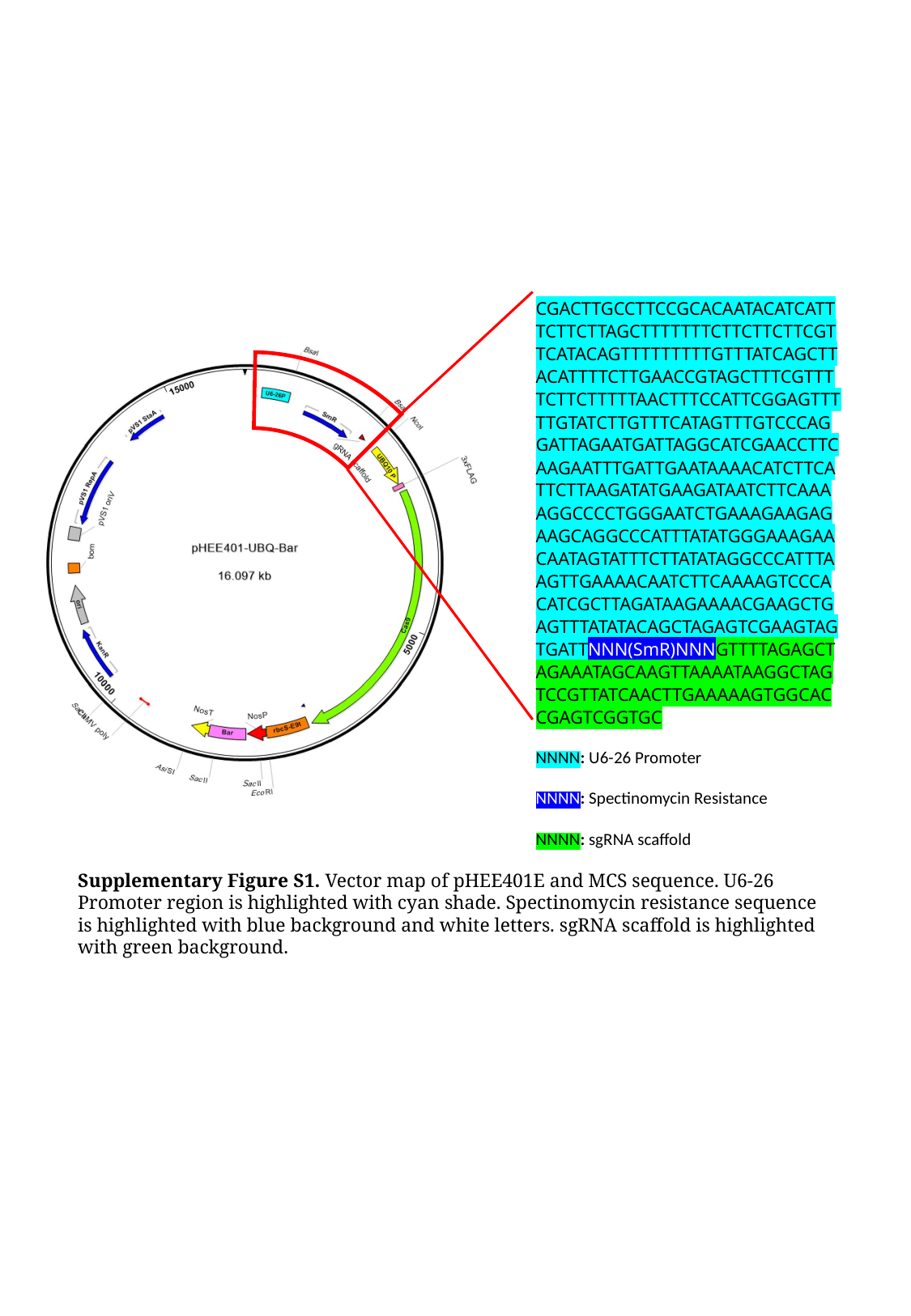

CGACTTGCCTTCCGCACAATACATCATTTCTTCTTAGCTTTTTTTCTTCTTCTTCGTTCATACAGTTTTTTTTTGTTTATCAGCTTACATTTTCTTGAACCGTAGCTTTCGTTTTCTTCTTTTTAACTTTCCATTCGGAGTTTTTGTATCTTGTTTCATAGTTTGTCCCAGGATTAGAATGATTAGGCATCGAACCTTCAAGAATTTGATTGAATAAAACATCTTCATTCTTAAGATATGAAGATAATCTTCAAAAGGCCCCTGGGAATCTGAAAGAAGAGAAGCAGGCCCATTTATATGGGAAAGAACAATAGTATTTCTTATATAGGCCCATTTAAGTTGAAAACAATCTTCAAAAGTCCCACATCGCTTAGATAAGAAAACGAAGCTGAGTTTATATACAGCTAGAGTCGAAGTAGTGATTNNN(SmR)NNNGTTTTAGAGCTAGAAATAGCAAGTTAAAATAAGGCTAGTCCGTTATCAACTTGAAAAAGTGGCACCGAGTCGGTGC
NNNN: U6-26 Promoter
NNNN: Spectinomycin Resistance
NNNN: sgRNA scaffold
Supplementary Figure S1. Vector map of pHEE401E and MCS sequence. U6-26 Promoter region is highlighted with cyan shade. Spectinomycin resistance sequence is highlighted with blue background and white letters. sgRNA scaffold is highlighted with green background.

## Slide 2
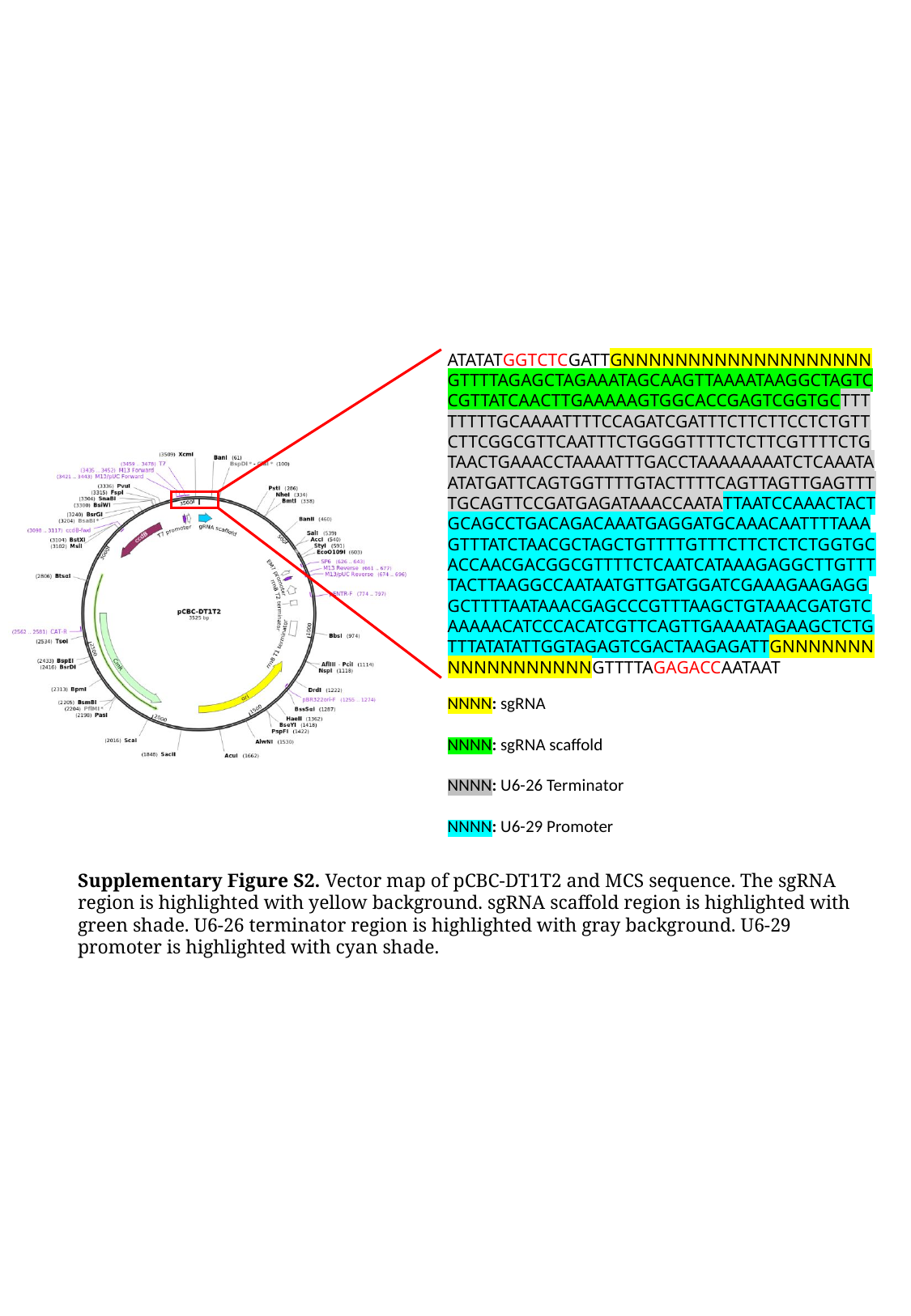

ATATATGGTCTCGATTGNNNNNNNNNNNNNNNNNNNGTTTTAGAGCTAGAAATAGCAAGTTAAAATAAGGCTAGTCCGTTATCAACTTGAAAAAGTGGCACCGAGTCGGTGCTTTTTTTTGCAAAATTTTCCAGATCGATTTCTTCTTCCTCTGTTCTTCGGCGTTCAATTTCTGGGGTTTTCTCTTCGTTTTCTGTAACTGAAACCTAAAATTTGACCTAAAAAAAATCTCAAATAATATGATTCAGTGGTTTTGTACTTTTCAGTTAGTTGAGTTTTGCAGTTCCGATGAGATAAACCAATATTAATCCAAACTACTGCAGCCTGACAGACAAATGAGGATGCAAACAATTTTAAAGTTTATCTAACGCTAGCTGTTTTGTTTCTTCTCTCTGGTGCACCAACGACGGCGTTTTCTCAATCATAAAGAGGCTTGTTTTACTTAAGGCCAATAATGTTGATGGATCGAAAGAAGAGGGCTTTTAATAAACGAGCCCGTTTAAGCTGTAAACGATGTCAAAAACATCCCACATCGTTCAGTTGAAAATAGAAGCTCTGTTTATATATTGGTAGAGTCGACTAAGAGATTGNNNNNNNNNNNNNNNNNNGTTTTAGAGACCAATAAT
NNNN: sgRNA
NNNN: sgRNA scaffold
NNNN: U6-26 Terminator
NNNN: U6-29 Promoter
Supplementary Figure S2. Vector map of pCBC-DT1T2 and MCS sequence. The sgRNA region is highlighted with yellow background. sgRNA scaffold region is highlighted with green shade. U6-26 terminator region is highlighted with gray background. U6-29 promoter is highlighted with cyan shade.

## Slide 3
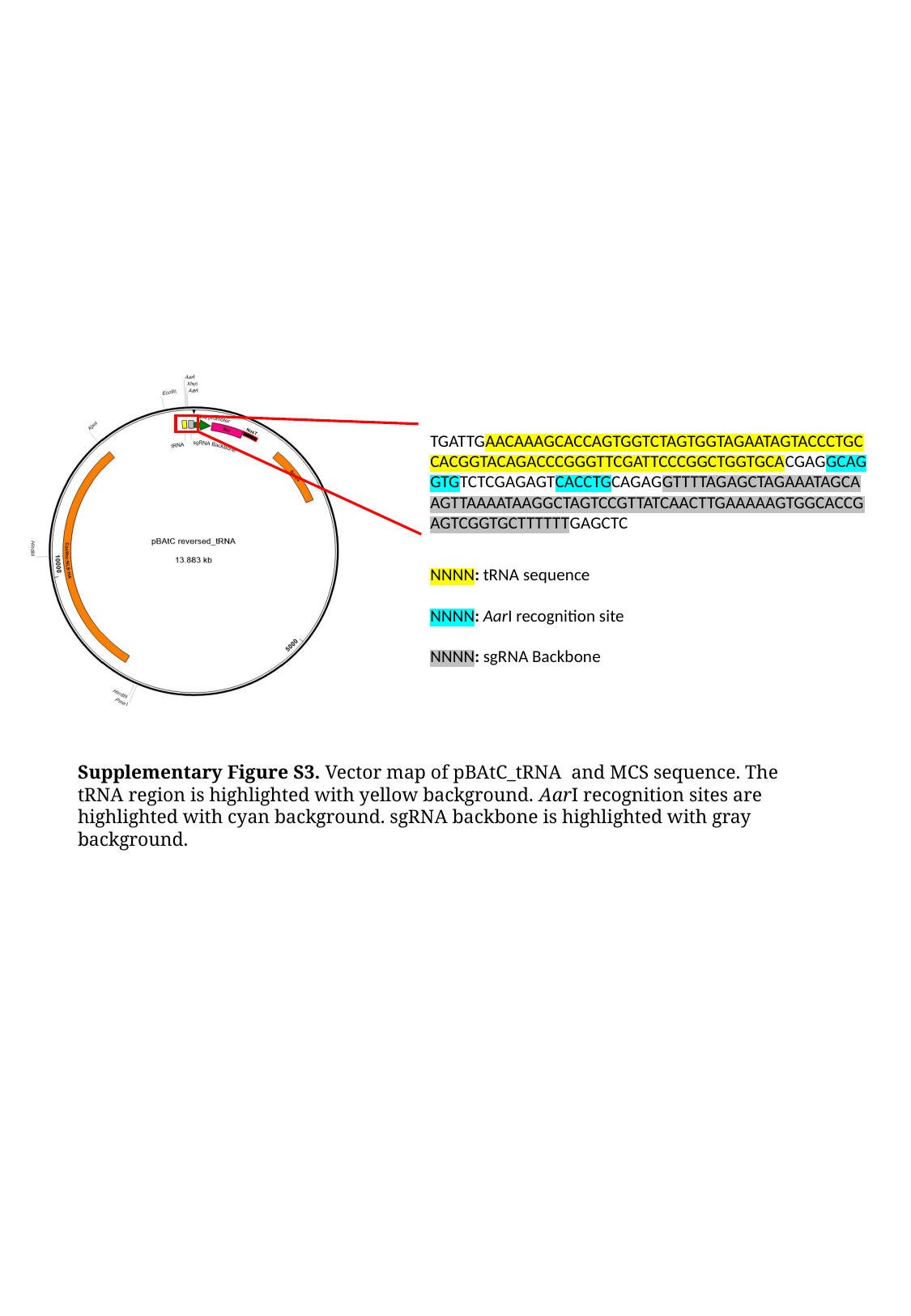

TGATTGAACAAAGCACCAGTGGTCTAGTGGTAGAATAGTACCCTGCCACGGTACAGACCCGGGTTCGATTCCCGGCTGGTGCACGAGGCAGGTGTCTCGAGAGTCACCTGCAGAGGTTTTAGAGCTAGAAATAGCAAGTTAAAATAAGGCTAGTCCGTTATCAACTTGAAAAAGTGGCACCGAGTCGGTGCTTTTTTGAGCTC
NNNN: tRNA sequence
NNNN: AarI recognition site
NNNN: sgRNA Backbone
Supplementary Figure S3. Vector map of pBAtC_tRNA and MCS sequence. The tRNA region is highlighted with yellow background. AarI recognition sites are highlighted with cyan background. sgRNA backbone is highlighted with gray background.

## Slide 4
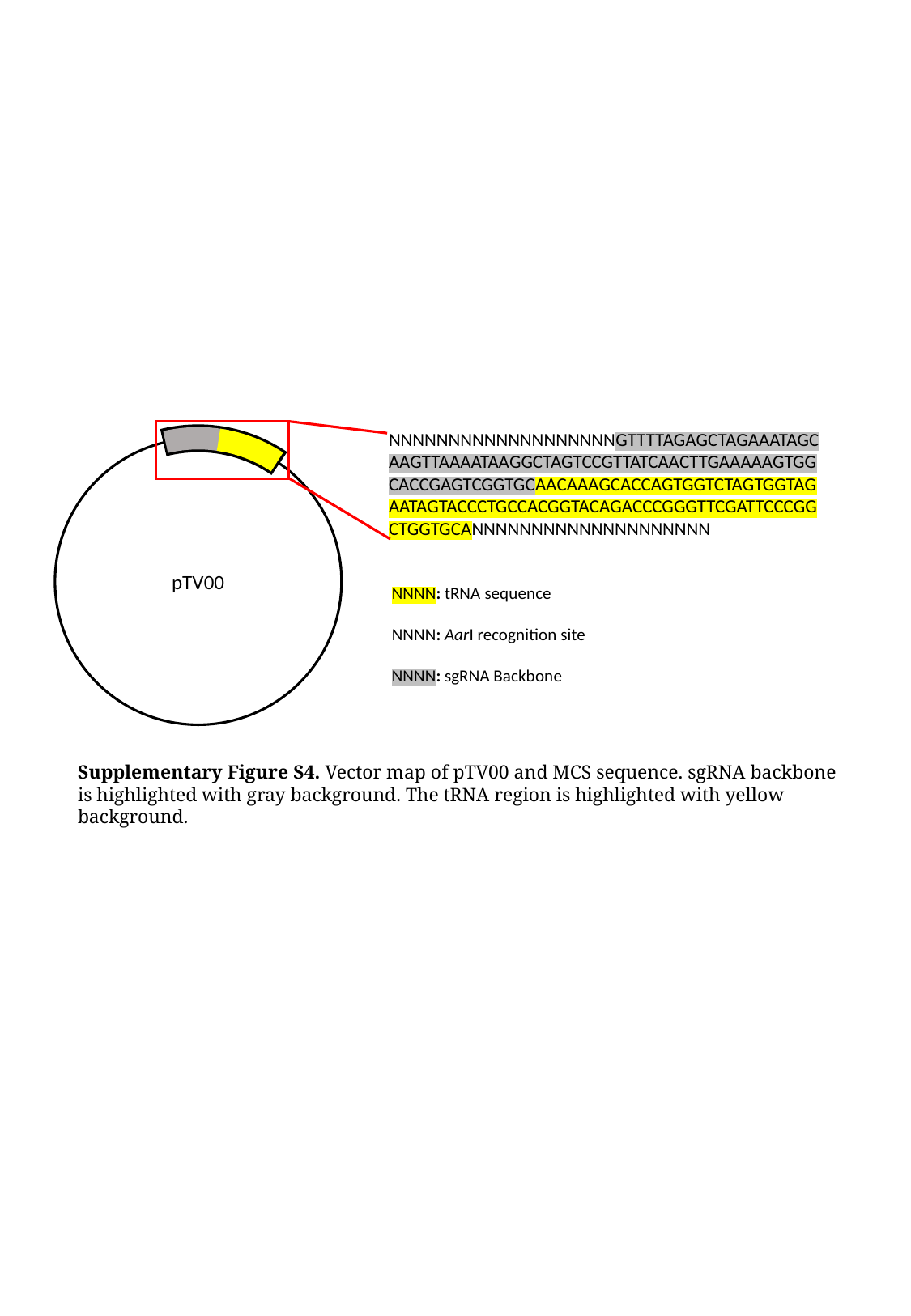

NNNNNNNNNNNNNNNNNNNGTTTTAGAGCTAGAAATAGCAAGTTAAAATAAGGCTAGTCCGTTATCAACTTGAAAAAGTGGCACCGAGTCGGTGCAACAAAGCACCAGTGGTCTAGTGGTAGAATAGTACCCTGCCACGGTACAGACCCGGGTTCGATTCCCGGCTGGTGCANNNNNNNNNNNNNNNNNNNN
pTV00
NNNN: tRNA sequence
NNNN: AarI recognition site
NNNN: sgRNA Backbone
Supplementary Figure S4. Vector map of pTV00 and MCS sequence. sgRNA backbone is highlighted with gray background. The tRNA region is highlighted with yellow background.

## Slide 5
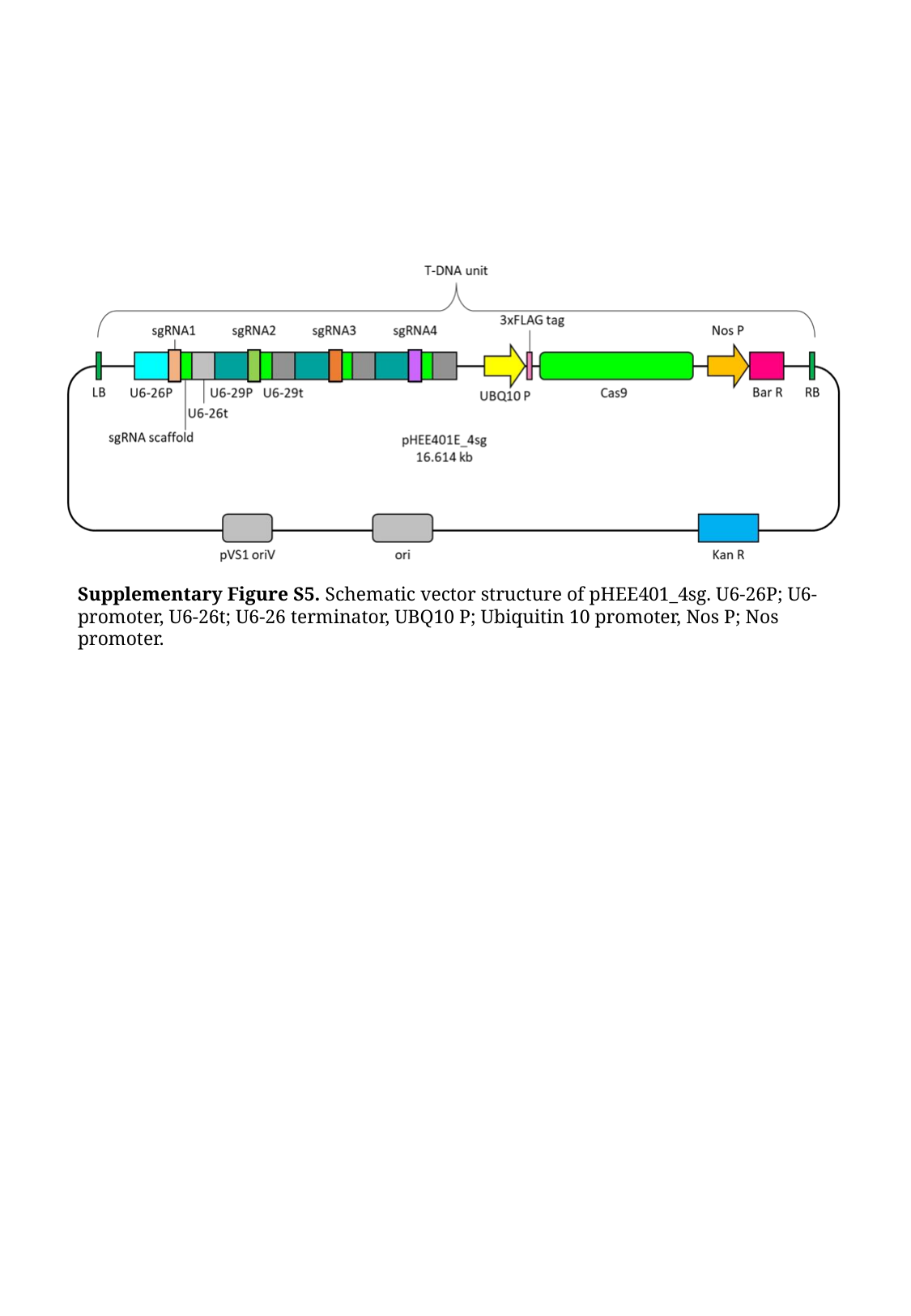

Supplementary Figure S5. Schematic vector structure of pHEE401_4sg. U6-26P; U6- promoter, U6-26t; U6-26 terminator, UBQ10 P; Ubiquitin 10 promoter, Nos P; Nos promoter.

## Slide 6
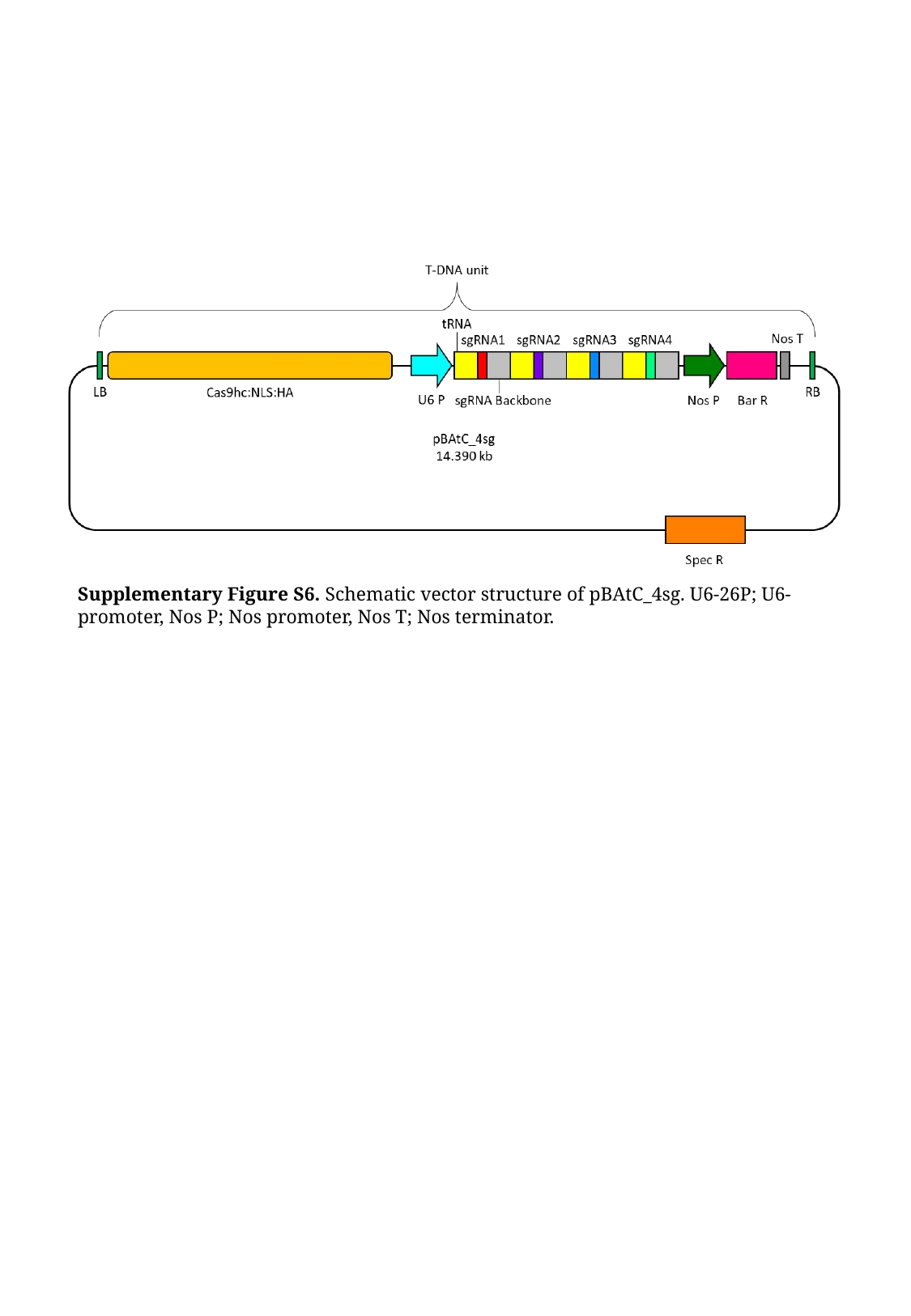

Supplementary Figure S6. Schematic vector structure of pBAtC_4sg. U6-26P; U6- promoter, Nos P; Nos promoter, Nos T; Nos terminator.

## Slide 7
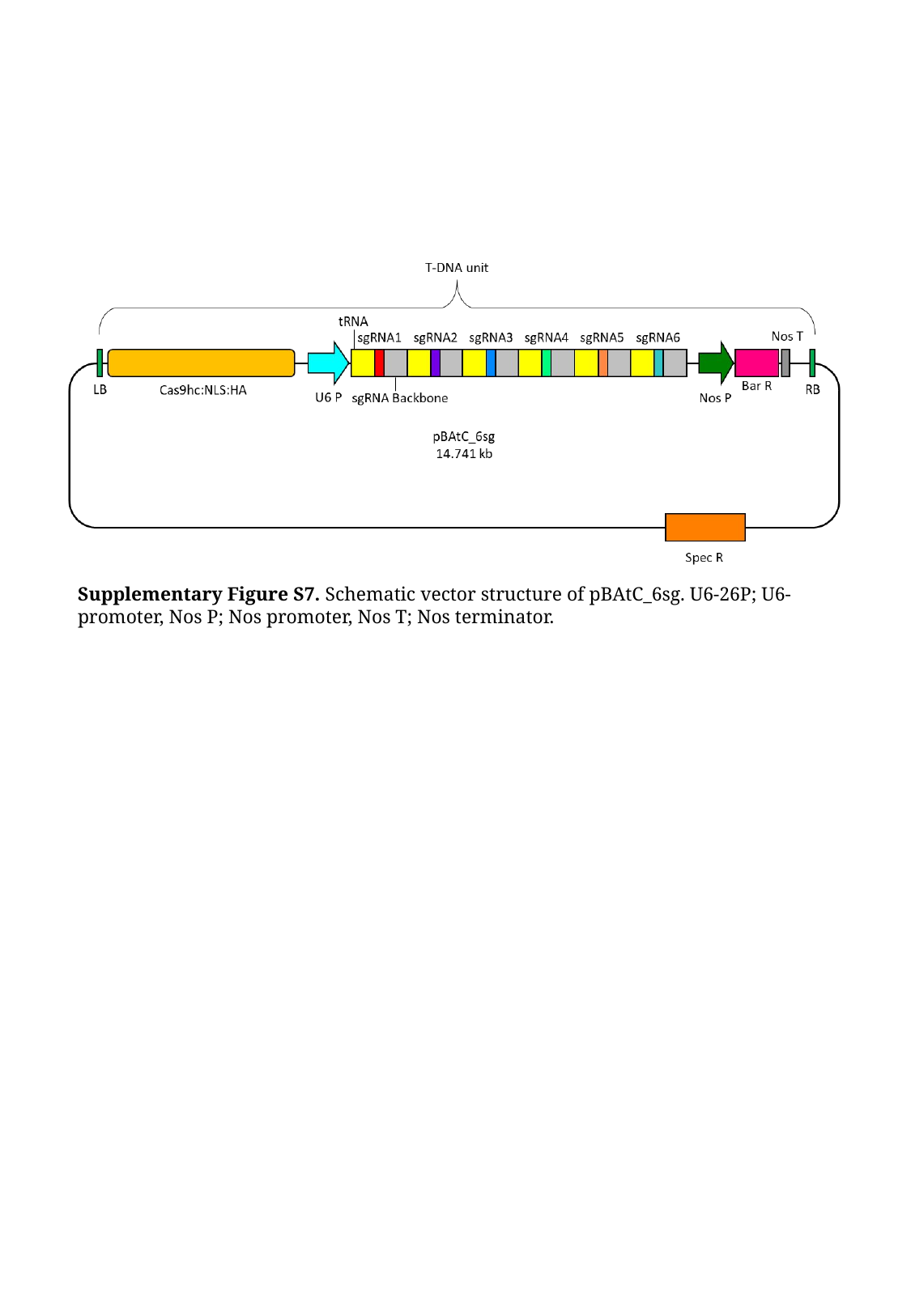

Supplementary Figure S7. Schematic vector structure of pBAtC_6sg. U6-26P; U6- promoter, Nos P; Nos promoter, Nos T; Nos terminator.
